# Alcohol-related cancer morbidity and mortality are stratified using modified albumin platelet product

**DOI:** 10.1038/s41598-023-50778-x

**Published:** 2024-01-11

**Authors:** Koji Fujita, Asahiro Morishita, Kyoko Oura, Masafumi Ono, Takashi Himoto, Tsutomu Masaki

**Affiliations:** 1 Department of Gastroenterology and Neurology, Faculty of Medicine, Kagawa University, Saiwai 1-1, Takamatsu, Kagawa 760-8521 Japan; 2 Department of Medical Technology, Kagawa Prefectural University of Health Sciences, 281-1 Hara, Mure, Takamatsu, Kagawa 761-0123 Japan

**Keywords:** Health care, Risk factors

## Abstract

Alcohol abuse is associated with several diseases, such as hepatocellular carcinoma, cirrhosis, and extrahepatic malignancies. Recently, we reported albumin platelet product (APP) and modified APP (mAPP) as novel indices of liver fibrosis staging and prognosis in patients without alcoholic liver diseases. This retrospective cohort study aimed to extend application of APP and mAPP in prognosis prediction of patients with alcoholic liver diseases. We enrolled 222 patients with alcoholic liver diseases based on their medical records. Cut-off values of APP = 4.349 and mAPP = 2.484 were adopted based on a past report. Hazard ratios of APP and mAPP were compared to those of albumin-bilirubin score and fibrosis-4 index. The primary and secondary endpoints were carcinogenesis and death, respectively. Thus, APP = 4.349 and mAPP = 2.484 significantly differentiated cancer-free survival and overall survival in univariate analysis. Hazard ratios of mAPP = 2.484 were greater than those of the albumin-bilirubin score of -2.270 and fibrosis-4 index of 3.25. Multivariate analysis revealed mAPP = 2.484 as an independent risk factor for carcinogenesis and overall death. In conclusion, mAPP is a simple index to stratify patient’s risk for carcinogenesis and death.

## Introduction

The harmful use of alcohol is a leading cause of more than 200 diseases and injury conditions, resulting in three million deaths annually, according to the World Health Organization. This represents 5.3% of all deaths (https://www.who.int/news-room/fact-sheets/detail/alcohol).

The liver is one of organs that can be severely impacted by harmful alcohol consumption. The long-term effects of alcohol on the liver can range from pure steatosis, liver fibrosis, and cirrhosis to hepatocellular carcinoma (HCC)^1^. Moreover, excessive alcohol intake has been associated with carcinogenesis in several organs in addition to the liver, such as the esophagus and oropharyngeal region^2^. Unlike cardiovascular diseases associated with alcohol abuse, there are no established thresholds for alcohol consumption regarding carcinogenesis^3,4^.

Previously, we reported that the albumin platelet product (APP), a simple byproduct of the serum albumin level and platelet count, indicates the staging of liver fibrosis, the stratification of HCC-free survival, and the overall survival of patients with chronic liver diseases, including hepatitis C and B viral infections (HCV and HBV, respectively), autoimmune hepatitis, and primary biliary cholangitis^5^. Furthermore, a modified version of APP (mAPP), a three-math construct constructed using serum albumin level, platelet count, and total bilirubin (T-Bil), is equivalent in its potential to diagnose advanced liver fibrosis and cirrhosis in the previous study. However, neither APP nor mAPP, had been validated in a cohort of patients with alcoholic liver diseases.

The present study aimed to extend the clinical application of APP and mAPP as prognostic indicators for patients with alcoholic liver diseases.

## Results

### Generation of a study cohort

A total of 419 patients were recruited based on diagnoses of alcoholic liver diseases (Supplementary Fig. 1); 81 were excluded owing to the presence of confounding factors, such as viral infection or autoimmune liver diseases, resulting in 338 patients with alcoholic liver diseases. After excluding 116 patients with malignancies at baseline or within 30 days of the diagnosis of alcoholic liver disease, the final study cohort consisted of 222 patients.

### Baseline characteristics of patients

The median age of the patients was 60 years, predominantly comprising men (Table 1). The median gamma-glutamyl transferase (GGTP) values were above the upper limit of the normal range for both male and female patients. The median platelet count was above 150 × 10^9^/L. The median APP and mAPP values exceeded their cut-off values for cirrhosis (above 4.349 and 2.484 for APP and mAPP, respectively).

**Table 1:** Baseline characteristics of patients.

	Median (IQR) or case number
Age	60 (48 to 68)
Male/female	174/48
Total protein (g/L)	72 (68 to 76)
Platelet count (10^9/L)	169 (116 to 215)
Albumin (g/L)	41 (36 to 44)
Creatinine (mg/dL)	0.71 (0.60 to 0.87)
Total bilirubin (mg/dL)	1.0 (0.7 to 1.5)
AST (U/L)	56 (33 to 101)
ALT (U/L)	40 (25 to 71)
ALP (U/L)	308 (236 to 416)
Gamma GTP (U/L)	196 (76 to 456)
Male	211 (78 to 499)
Female	164 (75 to 372)
HbA1c (%) < 6.2 / > 6.2	199/23
Prothrombin time (%)	84 (63 to 100)
PT-INR	1.09 (1.00 to 1.27)
APP	6.680 (3.862 to 9.301)
modified APP	3.824 (1.663 to 7.425)
ALBI score	-2.649 (-3.028 to -2.109)
Fibrosis-4 index	3.403 (1.905 to 5.867)
MELD score	9 (7 to 12)

APP, Albumin platelet product; ALBI score, albumin bilirubin score; IQR, interquartile range; MELD score, model for end-stage liver disease score.

### Patient follow-up

The patients were followed-up for a median of 1492 days, with a first quartile value of 615 days (Table 2). A total of 41 patients were diagnosed with complications of malignancies, 23 patients were complicated with HCC. Colon, gastric, prostate, and oropharyngeal cancers were diagnosed in four, three, three, and two patients, respectively. Moreover, cholangiocellular, esophageal, duodenal, lung, and thyroid cancer and parotid tumor were diagnosed in one patient each. The median time from baseline to carcinogenesis was 1819 days. A total of 29 patients died during the follow-up period. Among them, 12 had cirrhosis, and eight had HCC due to alcoholic cirrhosis. The median time from baseline to death was 1937 days.

**Table 2:** Follow-up information.

	Median (IQR) or case number
Observation period (day)	1492 (651 to 2902)
Time to carcinogenesis (day)	1819 (750 to 2711)
Time to death (day)	1937 (859 to 3484)
Carcinogenesis	41
Hepatocellular/cholangiocellular carcinoma	23/1
Colon/gastric/esophageal/duodenal cancer	4/3/1/1
Prostate cancer	3
Oropharyngeal cancer	2
Others (lung/thyroid/parotid)	3 (1 /1 /1)
Death	29
Cirrhosis	12
Hepatocellular carcinoma	8
Gastric/esophageal/common bile duct/Pancreatic cancer	1/1/1/1
Duodenal hemorrhage	1
Primary amyloidosis	1
Unknown	3

IQR, interquartile range.

### Univariate analysis for prognosis prediction

**Figure 1:**
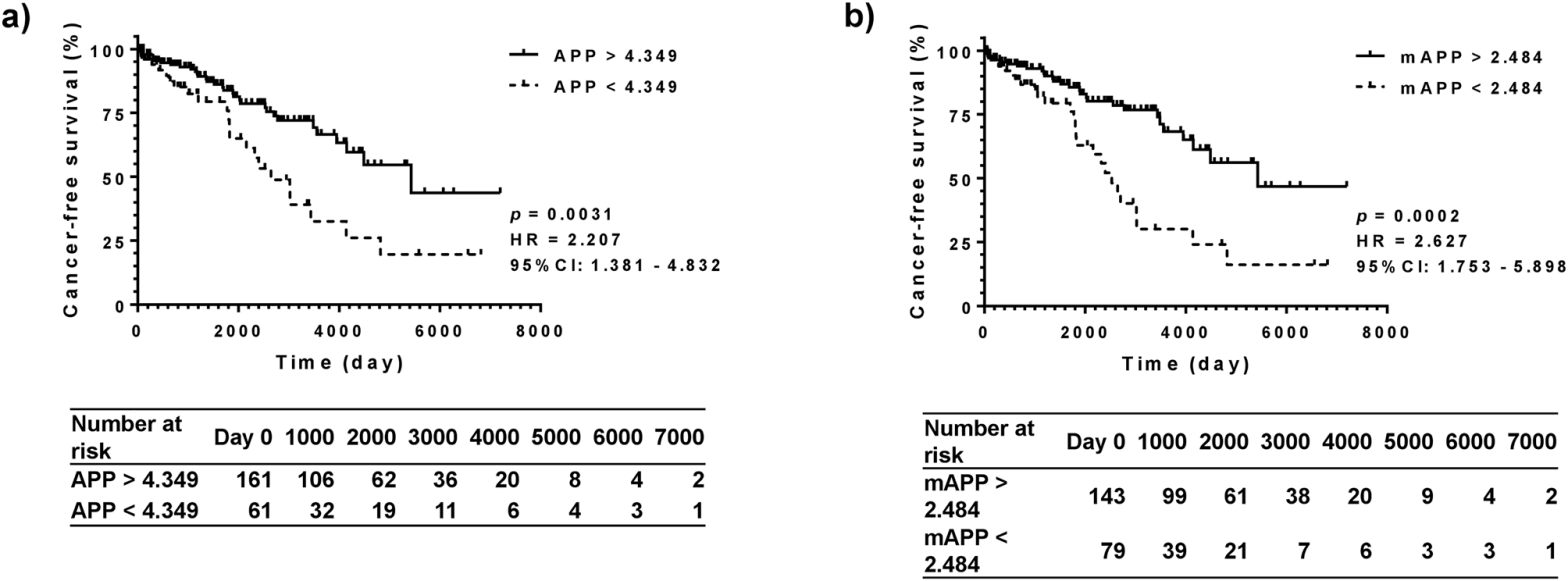
Cancer-free survival evaluated using APP and mAPP. A univariate analysis was performed to evaluate the prognostic potential of APP and modified APP (mAPP) for cancer-free survival. APP = 4.349 and mAPP = 2.484 were adopted as cut-off values. (**a**) APP = 4.349 significantly differentiates the cancer-free survival of patients with alcoholic liver diseases with an HR of 2.207 (*P* = 0.0031). (**b**) mAPP was able to predict the prognosis of the patients with a better HR of 2.627 than that of APP (*P* = 0.0002). HCC, hepatocellular carcinoma; HR, hazard ratio; mAPP, modified albumin platelet product.

A univariate analysis was conducted to determine the prognostic abilities of APP and mAPP, compared to that of the albumin-bilirubin (ALBI) score and fibrosis-4 (FIB-4) index. Both APP = 4.349 and mAPP = 2.484 significantly stratified the cancer-free survival prognosis of patients, with hazard ratios of 2.207 (*P* = 0.0031) and 2.627 (*P* = 0.0002) (Fig. 1). Compared to that of APP, mAPP had a better hazard ratio. The ALBI score of − 2.270 and FIB-4 index of 3.25 were able to significantly stratify the survival curves with lower hazard ratios (1.923 for ALBI = − 2.270, *P* = 0.0171; 2.181 for FIB-4 = 3.25, *P* = 0.0046) than that of mAPP (Supplementary Figs. 2a, 4a).

**Figure 2:**
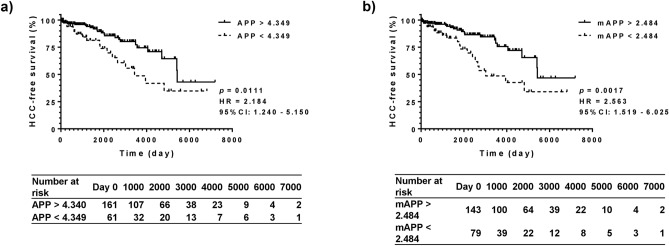
HCC-free survival stratified using APP and mAPP. Focusing on hepatocellular carcinoma (HCC), differentiation of HCC-free survival was tested using APP and modified APP (mAPP). Both patients with APP > 4.349 (HR, 2.184; *P* = 0.0111) (**a**) and with mAPP > 2.484 (HR, 2.563; *P* = 0.0017) (**b**) showed a better overall survival compared to others. The hazard ratios were better for mAPP than for APP. HCC, hepatocellular carcinoma; HR, hazard ratio; mAPP, modified albumin platelet product.

In addition, predictive values of HCC-free survival were compared among APP, mAPP, ALBI score, and FIB-4. As shown in Fig. 2a,b, APP and mAPP significantly stratified the cohort into two groups, a group with better HCC-free survival and the worse one. The hazard ratios were 2.184 for APP (*P* = 0.0111) and 2.563 for mAPP (*P* = 0.0017). An ALBI score of − 2.270 and a FIB-4 index of 3.25 resulted in lower hazard ratios of 1.985 (*P* = 0.0282) and 2.264 (*P* = 0.0120), compared to that of mAPP = 2.484 (Supplementary Figs. 2b, 4b).

**Figure 3:**
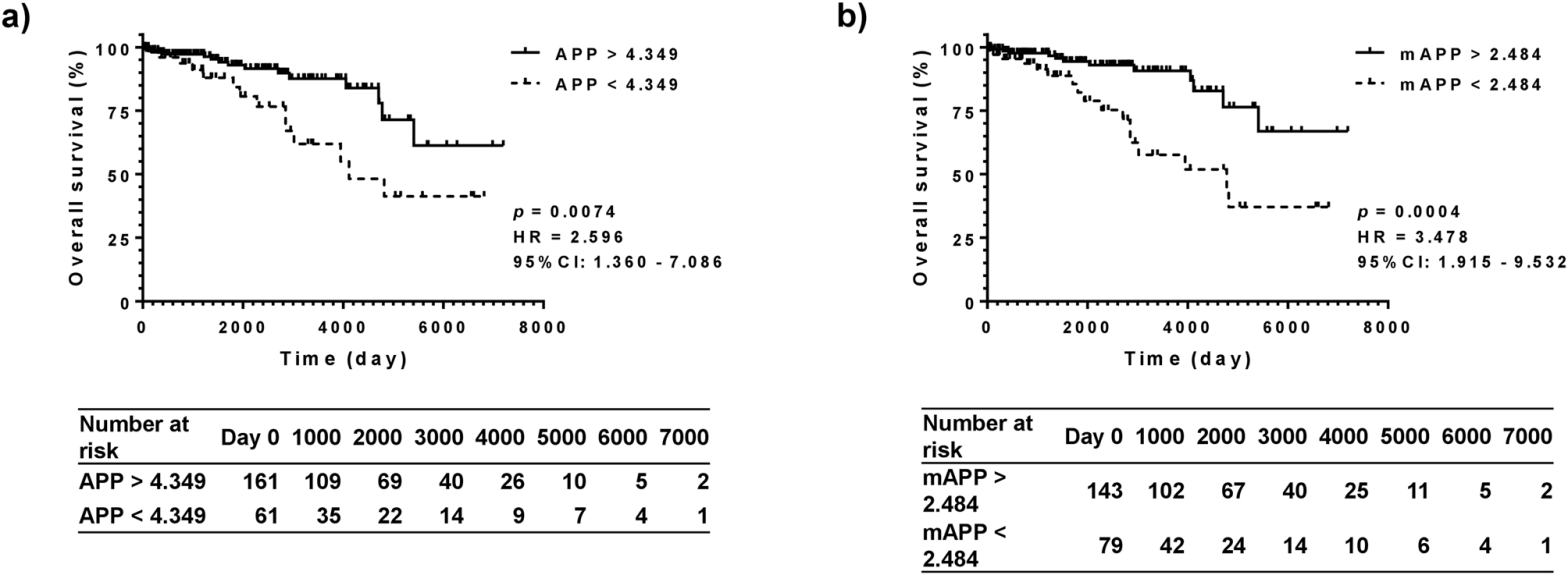
Overall survival evaluated using APP and mAPP. The prognostic potential of APP and modified APP (mAPP) for overall survival was determined using univariate analysis. (**a**) Patients with APP > 4.349 presented a better overall survival compared to those of patients with APP < 4.349 with an HR of 2.596 (*P* = 0.0074). (**b**) An HR of mAPP = 2.484 (3.478; *P* = 0.0004) for overall survival was better than that of APP = 4.349. APP, albumin platelet product; APP, albumin platelet product; HCC, hepatocellular carcinoma; HR, hazard ratio; mAPP, modified albumin platelet product.

Overall survival was also significantly differentiated using APP and mAPP (Fig. 3). Similar to the analysis of cancer-free survival, mAPP (HR, 3.478; *P* = 0.0004) was better than APP (HR, 2.596; *P* = 0.0074), with a higher hazard ratio. The ALBI score and FIB-4 also distinguished overall survival, but the hazard ratios of the ALBI score of − 2.270 (HR, 2.710; *P* = 0.0050) and FIB-4 index of 3.25 (HR, 2.696; *P* = 0.0127) were lower than that of mAPP (Supplementary Figs. 3, 5).

Consequently, a mAPP score of 2.484 was the best among APP, mAPP, ALBI grade, and FIB-4 index for stratifying cancer-free, overall, and HCC-free survival rates. Therefore, we focused on mAPP for further evaluation.

### Multivariate analysis for prognosis prediction

A multivariate analysis was conducted to determine whether mAPP was an independent risk factor for both carcinogenesis and deaths after adjusting for age, sex, and hemoglobin A1c (HbA1c) (%) using a Cox proportional hazard model. As shown in Table 3, mAPP was an independent risk factor for all endpoints, including carcinogenesis (HR, 2.477; *p* = 0.0072), carcinogenesis or death (HR, 3.244; *p* = 0.0003), and overall death (HR, 3.622; *p* = 0.0007). Age was significantly associated with an increased risk of carcinogenesis (HR, 1.058; *p* = 0.0003), and of carcinogenesis or death (HR, 1.046; *p* = 0.0005). However, age was not a significant variable for predicting overall mortality (*p* > 0.05).

**Table 3:** Multivariate analysis for carcinogenesis and mortality.

		Age	Male/female	mAPP < 2.484 / > 2.484	HbA1c > 6.2%	Harrell’s C index (SE)
Carcinogenesis	Hazard ratio	1.058	1.538	2.477	2.352	0.699 (0.058)
	95% CI	1.026 to 1.091	0.538 to 4.399	1.278 to 4.802	1.121 to 4.933	
	*P*-value	0.0003	0.4219	0.0072	0.0236	
Carcinogenesis or deaths	Hazard ratio	1.046	1.023	3.244	2.283	0.690 (0.044)
	95% CI	1.020 to 1.073	0.475 to 2.205	1.862 to 5.652	1.176 to 4.429	
	*P-*value	0.0005	0.9535	0.0003	0.0147	
Overall deaths	Hazard ratio	1.031	0.873	3.622		0.707 (0.046)
	95% CI	0.996 to 1.066	0.329 to 2.319	1.718 to 7.634	N/A	
	*P*-value	0.0835	0.7857	0.0007		

95% CI, 95% confidence interval; mAPP, modified Albumin platelet product; N/A, not applicable; SE, standard error.

HbA1c > 6.2% also acted as an independent risk factor for the outcomes when an endpoint was put at carcinogenesis (HR, 2.352; *p* = 0.0236), or at carcinogenesis or death (HR, 2.283; *p* = 0.0147). HbA1c was not included in a multivariate analysis for overall death according to a guideline^6^. A similar analysis for HCC complication was omitted for the same rationale^6^. Harrell’s C-index was approximately 0.7 for all calculations.

### GGTP reduction rate from baseline to outcome

To estimate whether habitual alteration of alcohol consumption correlated with patient outcomes, the GGTP reduction rate from baseline to outcome was calculated. In 41 patients with carcinogenesis during the observation period, the GGTP values at diagnosis of malignancies were compared to those at baseline. In 167 patients whose observation was censored alive with no malignancies during the observation period, GGTP at censor was compared to that at baseline. As shown in Supplementary Fig. 6, the GGTP levels were more reduced in patients without malignancies compared to those of patients complicated with malignancies, as a trend (*p* > 0.05).

## Discussion

The current study was conducted to investigate the potential of APP and mAPP for managing patients with alcoholic liver diseases. APP and mAPP were established based on an analysis of patients with non-alcoholic etiologies. The present study revealed that (1) APP and mAPP can stratify cancer-free survival and overall survival, with mAPP showing better performance, and that (2) mAPP is an independent risk factor for carcinogenesis and deaths.

Alcohol is considered the most severe carcinogen by the International Agency for Research on Cancer since 1988 (https://publications.iarc.fr/Book-And-Report-Series/Iarc-Monographs-On-The-Identification-Of-Carcinogenic-Hazards-To-Humans/Alcohol-Drinking-1988). Among newly diagnosed cancers worldwide, an estimated 4.1% of cancers (741,300 cases) in 2020 were attributed to alcohol consumption^7^. Alcoholic beverages consumption is related to cancers in specific organs, including the oral cavity, pharynx, larynx, esophagus, and liver. The prevalence of colorectal cancer, female breast cancer, and pancreatic cancer also increase in accordance with alcohol intake.

Alcohol drives carcinogenesis via several pathways, including acetaldehyde production, excess oxidative stress, alteration of gut microbiome, and dysregulated retinoid metabolism^8^. Acetaldehyde, a metabolites of ethanol, has affinity to DNA. Acetaldehyde directly binds to DNA and may change DNA synthesis and repair^9^. Acetaldehyde results in DNA methylation through inhibition of DNA methyltransferase^9^. The process of oxidizing ethanol to acetaldehyde causes oxidative stress, which provides the basis of carcinogenesis^10^. Ethanol metabolism by CYP2E1 produces excess reactive oxygen species known as superoxide anion and hydrogen peroxide, which contributes to liver, colorectal, and esophageal carcinogenesis^11,12^. Liver cancer will be initiated by habitual alcohol consumption partially via altered gut microbiome in the context of gut-liver axis^13^. Chronic alcohol intake reduces the blood retinol levels, which enhances carcinogenesis in the head and neck regions^14^.

APP and mAPP were initially established using a training cohort of patients with a biopsy-proven staging of fibrosis^5^, with a cut-off value for cirrhosis determined as 4.349 and 2.484 for APP and mAPP, respectively. The cut-off value of APP stratified HCC-free survival and overall survival. A validation cohort confirmed that APP was able to diagnose cirrhosis and predict the prognosis of patients. However, the training and validation cohorts comprised patients with non-alcoholic liver diseases. Therefore, we aimed to extend the application of APP and mAPP to patients with alcoholic liver diseases.

Alcoholic liver diseases have a spectrum from simple steatosis to hepatitis and cirrhosis^15^. The current cohort may have presented with mild alcoholic changes in the liver based on their baseline platelet count and chemistry panel^16^. As described in a clinical guideline as an early alcoholic liver disease, patients in the current study were characterized with minimal elevation of AST and ALT, elevated GGTP with normal T-Bil levels, and prothrombin time (PT)-international normalized ratio (INR)^17^. Most patients had a model for end-stage liver disease (MELD) score of < 10 points at baseline (Table 2). Thus, evaluation of baseline liver function using Maddrey’s discrimination function was omitted in the current study^18,19^.

However, a high proportion of patients with histologically confirmed alcoholic liver diseases may remain asymptomatic and show no laboratory abnormalities^20^. In the current cohort, approximately 10% of the patients died from cirrhosis or HCC within the observational period, as shown in Table 2. The around 10% of liver-related mortality in the current observation was orchestrated with a past research^1^. Our findings suggest that mAPP can effectively stratify the prognosis of patients, even those in the early stages of alcoholic liver diseases.

In a multivariate analysis with endpoints of carcinogenesis, and carcinogenesis or death, HbA1c > 6.2% was extracted as an independent risk factor accompanied with mAPP > 2.484 (Table 3). Diabetes mellitus is an established risk factor for cancer prevalence in several organs^21^. According to a past report, alcohol abusers tend to be complicated with diabetes mellitus^22^. Habitual drinking will clinically cause chronic pancreatitis, resulting in impaired insulin secretion from pancreatic beta cells^23^.

mAPP has several strengths, including the incorporation of serum albumin in its equation. Serum albumin exhibits a stronger association with the prognosis of patients with alcoholic liver diseases compared to the association exhibited by pathological findings in liver tissues^24^. The second strength of mAPP is that it considers platelet count in its calculation. As the decrease in platelet count correlates with liver fibrosis progression and portal hypertension, mAPP can detect liver fibrosis more sensitively than ALBI grade can^25^. Furthermore, alcohol may induce thrombocytopenia by transiently regulating the spleen’s store of platelets^26^. The third strength lies its simple equation excluding natural logarithm or square root, compared to the ALBI score and FIB-4 index.

A limitation of the current study might be that patients were selected from a tertiary hospital, where a relatively limited number of alcoholic patients were referred from clinics and hospitals in the community. Therefore, the clinical application of mAPP in patients with alcoholic liver diseases should be validated using a more general population of alcohol abusers. In addition, the cut-off values of APP, mAPP, ALBI score, and FIB-4 index were derived from the cut-off values got cirrhosis of different etiologies: APP = 4.349 and mAPP = 2.484 pertain to a mainly HCV infection, an ALBI score = − 2.270 pertains to HCV infection, and an FIB-4 index = 3.25 pertains to non-alcoholic steatohepatitis. Optimal cut-off values for prognosis, not for cirrhosis, should be further searched in patients with alcoholic liver diseases based on a larger cohort. Finally, accumulated doses of alcohol consumption over time were not incorporated into the study. Alteration of the GGTP levels between the baseline and outcome was considered as a surrogate for it.

In conclusion, mAPP can predict cancer-free and overall survival. The simple index can serve as a convenient measure for managing patients with alcohol abuse.

## Methods

### Ethics

This study was conducted in accordance with the ethical principles of the Declaration of Helsinki and was approved by the Institutional Review Board of Kagawa University, Faculty of Medicine (Heisei-30-151)^27^. Informed consent was obtained from all patients for the analysis of their clinical data. In cases where patients had died and had no listed relatives in their medical records, we provided opt-out methods for the relatives of the dead participants by publishing a summary of this study on our university website^28,29^.

### Study design

The current study was a retrospective observational study conducted at an outpatient clinic of internal hepatology at a single university hospital. The baseline of observation was established at the time of the diagnosis of alcoholic liver disease. The primary endpoint was the occurrence of malignancies in any organ, except for carcinomas in situ of the gastrointestinal tract. The secondary endpoint was defined as death from any cause. Cut-off values for APP and mAPP were determined based on the diagnostic thresholds for cirrhosis in patients with non-alcoholic etiologies, as reported in a previous study (APP = 4.349 and mAPP = 2.484 for cirrhosis)^5^.

To compare the diagnostic abilities of the two indices with the ALBI grade, we adopted a cut-off value between ALBI grades 2a and 2b (ALBI score = − 2.270)^30^. An ALBI score of − 2.270 was an approximation of a cut-off ALBI score = − 2.125 for cirrhosis in patients with hepatitis C infection^31^. For FIB-4, a cut-off value of 3.25 was employed as an indicator of high risk of cirrhosis, as reported in a past study^32^.

HbA1c of 6.2% was determined as a threshold of impaired glucose tolerance with or without diabetes treatment. In patients with no record of HbA1c, their HbA1c values were assumed to be < 6.2%. The study was conducted in accordance with the STROBE statement^33^.

### Patients

Patients were recruited based on a medical record. Patients who were diagnosed as alcoholic liver diseases, including alcoholic steatosis, alcoholic hepatitis, or alcoholic cirrhosis, between January 1, 2002 and December 31, 2021 were registered. After reviewing their clinical data, patients with alcoholism were included in the study, while those with any confounding factors at presentation were excluded. Specifically, patients with hepatitis viral infection, autoimmune hepatitis, or other specific etiologies were excluded. Patients with HCV were defined as those with a history of positive HCV-RNA or anti-viral therapy. Patients with spontaneous remission of HCV, defined as those with positive anti-HCV antibodies, negative HCV-RNA, or no history of viral eradication therapy, were included in the current cohort. Patients with HBV infection were defined as those positive for HBV surface (HBs) antigen and HBV-DNA, while those with functional cure without any history of anti-viral therapy were included. The functional cure was defined as a negative status for HBs antigen or HBV-DNA and a positive status for HBV core (HBc) and HBs antibody. Patients with autoimmune hepatitis were identified based on the criteria of International Autoimmune Hepatitis Group scores ≥ 10.

Patients with malignancies at the diagnosis of alcoholic liver diseases were excluded, whereas malignancies were defined as all malignant diseases except for carcinomas in situ of the gastrointestinal tract. Patients who were diagnosed with malignancies within 30 days after the diagnosis of alcoholic liver diseases were also excluded.

### Clinical data

The following clinical data were extracted from the patient’s medical records: age, sex, history of alcohol consumption, and medications. For laboratory data, platelet count, aspartate aminotransferase, alanine aminotransferase, alkaline phosphatase, GGTP, T-Bil, HbA1c (%), and PT-INR. T-Bil (mg/dL) was converted to T-Bil (µmol/L) according to the following equation: T-Bil (mg/dL) × 17.2. HbA1c (%) as described by Japan Diabetes Society (JDS) was converted to values based on the National Glycohemoglobin Standardization Program (NGSP)^34^.

APP was calculated using the following equation: albumin (g/L) × platelet (10^9^/L)/1000, and mAPP was calculated using the following equation: albumin (g/L) × platelet (10^9^/L)/(T-Bil (µmol/L) × 100)^5^. The ALBI score was calculated based on a calculation from a previous report: log_10_ T-Bil (µmol/L) × 0.66 + albumin (g/L) ×  (− 0.085)^35^. FIB-4, a conventional liver fibrosis index, was calculated using the following equation: age × AST (U/L)/(Plt (10^9^/L) × √ALT (U/L))^36^. MELD score was derived according to the equation: 9.57 × ln (serum creatinine, mg/dL) + 3.78 × ln (T-Bil, mg/dL) + 11.20 × ln (PT-INR) + 6.43 × Constance for etiology^37^. The constance for etiology was set at zero in the current cohort.

### Statistical analyses

Continuous variables were presented as medians and interquartile ranges and analyzed using the Mann–Whitney *U* test or Spearman’s rank correlation coefficient. The statistical analyses above were performed using GraphPad Prism 6 (GraphPad Software, La Jolla, CA). A Cox proportional hazard model was analyzed using EZR (Saitama Medical Center, Jichi Medical University, Saitama, Japan), a graphical user interface for R software (The R Foundation for Statistical Computing, Vienna, Austria)^38,39^. The number of variables in a multivariate analysis was controlled following a rule that number of events should be as 10 times as number of variables in proportional hazard ratio model^6^. *P* < 0.05 was considered statistically significant.

### Supplementary Information

Supplementary Legends.

Supplementary Figure 1.

Supplementary Figure 2.

Supplementary Figure 3.

Supplementary Figure 4.

Supplementary Figure 5.

Supplementary Figure 6.

## Data Availability

The data that support the findings of this study are available from the corresponding author upon reasonable request.
