# Supplementary material for: Alcohol-related cancer morbidity and mortality are stratified using modified albumin platelet product
Source: Sci Rep. 2024 Jan 11;14:1052. doi: 10.1038/s41598-023-50778-x (PMC10781945; doi:10.1038/s41598-023-50778-x)
Supplement: Supplementary file 1 — Supplementary Legends. [file 41598_2023_50778_MOESM1_ESM.docx]

**Supplementary Fig. 1. Diagram of the patient selection process in the current cohort**

A total of 419 patients had alcoholic liver diseases as per their medical records. After reviewing clinical information, 81 patients revealed confounding factors, including viral infections and autoimmune liver diseases. Excluding 116 patients with any malignant diseases at baseline or within 30 days from baseline, 222 patients were determined to be participants in the current study.

**Supplementary Fig. 2. Cancer-free and HCC-free survival prediction using ALBI grade**

ALBI grade was adopted in the stratification of the prognosis of patients for comparison with those using APP and modified APP (mAPP). A cut-off value of -2.270 for the ALBI score was adopted from that between ALBI grades 2a and 2b. (**a**) Cancer-free survival was significantly stratified using the ALBI score of -2.270 with an HR of 1.923 (*P*=0.0171), which was smaller than that of mAPP. (**b**) ALBI score of -2.270 also differentiated HCC-free survival with a hazard ratio of 1.985 (*P*=0.0282), which was smaller than that of mAPP.

ALBI, albumin-bilirubin; APP, albumin platelet product; HR, hazard ratio; mAPP, modified albumin platelet product.

**Supplementary Fig. 3. Overall survival evaluated using ALBI grade**

The ALBI score of -2.270 predicted the overall survival of the patients with an HR of 2.710 (*P*=0.0050), which was smaller than that of mAPP.

ALBI grade (score), albumin-bilirubin grade (score); HCC, hepatocellular carcinoma; HR, hazard ratio;

**Supplementary Fig. 4. Cancer-free and HCC-free survival prediction using the FIB-4 index**

Classifying patients using FIB-4 index of 3.25, survival curves were calculated for cancer-free and HCC-free survival. (**a**) Cancer-free survival yielded a hazard ratio of 2.181 (*P*=0.0046) between FIB-4 index <3.25 and >3.25. (**b**) Patients with FIB-4 indices <3.25 resulted in a better HCC-free survival compared to those of patients with FIB-4 indices >3.25, with a hazard ratio of 2.264 (*P*=0.0120).

FIB-4, fibrosis-4 index; HCC, hepatocellular carcinoma; HR, hazard ratio

**Supplementary Fig. 5. Overall survival evaluated using the FIB-4 index**

The FIB-4 index of 3.25 predicted the overall survival of the patients with an HR of 2.696 (*P=*0.0127), a lower value compared to that of mAPP.

FIB-4, fibrosis-4 index; HR, hazard ratio; mAPP, modified albumin platelet product

**Supplementary Fig. 6. GGTP reduction ratio from the baseline to the outcomes**

The GGTP reduction ratio was compared between the two cohorts; the first cohort comprised patients censored alive without malignancies through the observation (n=167); the other included patients complicated with malignancies (n=41). The GGTP reduction ratio (at outcome / baseline) was lower in patients censored without malignancies compared to those of patients complicated with malignancies as a tendency (*p* >0.05), based on the Mann–Whitney *U* test.

GGTP, gamma-glutamyl transferase
